# Zeaxanthin Dipalmitate Therapeutically Improves Hepatic Functions in an Alcoholic Fatty Liver Disease Model through Modulating MAPK Pathway

**DOI:** 10.1371/journal.pone.0095214

**Published:** 2014-04-16

**Authors:** Jia Xiao, Jiteng Wang, Feiyue Xing, Tao Han, Rui Jiao, Emily C. Liong, Man-Lung Fung, Kwok-Fai So, George L. Tipoe

**Affiliations:** 1 Department of Immunobiology, Institute of Tissue Transplantation and Immunology, Jinan University, Guangzhou, China; 2 School of Fishery, Zhejiang Ocean University, Zhoushan, China; 3 Department of Food Science and Engineering, Jinan University, Guangzhou, China; 4 GMH Institute of Central Nervous System Regeneration, Jinan University, Guangzhou, China; 5 Department of Anatomy, Li Ka Shing Faculty of Medicine, The University of Hong Kong, Hong Kong SAR, China; 6 Department of Physiology, Li Ka Shing Faculty of Medicine, The University of Hong Kong, Hong Kong SAR, China; 7 Brain Hormone Healthy Aging Centre, Li Ka Shing Faculty of Medicine, The University of Hong Kong, Hong Kong SAR, China; 8 Department of Infectious Diseases, Shenzhen Third People's Hospital, Shenzhen, China; Bambino Gesu' Children Hospital, Italy

## Abstract

In the current study, the therapeutic effects of zeaxanthin dipalmitate (ZD) on a rat alcoholic fatty liver disease (AFLD) model were evaluated. After-treatment with ZD from the 5^th^ week to the 10^th^ week in a 10-week ethanol intragastric administration in rats significantly alleviated the typical AFLD symptoms, including reduction in rat body weight, accumulation of hepatic fat droplets, occurrence of oxidative stress, inflammation, chemoattractive responses and hepatic apoptosis in the liver. The reduction of liver function abnormalities by ZD was partly through lower expression level of cytochrome P450 2E1 (CYP2E1), diminished activity of nuclear factor kappa B (NF-κB) through the restoration of its inhibitor kappa B alpha (IκBα), and the modulation of MAPK pathways including p38 MAPK, JNK and ERK. ZD treatment alone did not pose obvious adverse effect on the healthy rat. In the cellular AFLD model, we also confirmed the inhibition of p38 MAPK and ERK abolished the beneficial effects of ZD. These results provide a scientific rationale for the use of zeaxanthin and its derivatives as new complementary agents for the prevention and treatment of alcoholic liver diseases.

## Introduction

The abuse of alcohol is a severe social problem in the world with heavy burden in both health and economics. As one of the most prevalent liver diseases caused by alcohol over-consumption, alcoholic fatty liver disease (AFLD) affects over 2 million people in the U.S. (U.S. Census Bureau, Population Estimates, 2004). In China, it is estimated that 2.8% of population has AFLD or suspected AFLD [1]. The prevalence of AFLD is influenced by many factors, not only the abuse of alcohol, but also the gender discrepancy, genetic defects and other environmental factors, since only 1 in 5 heavy drinkers develops alcoholic hepatitis [2]. In the past decade, significant progress has been achieved to understand the molecular and cellular pathological mechanisms contributing to AFLD. The spectrum of AFLD ranges from simple steatosis (fat accumulation without inflammatory infiltration) to alcoholic steatohepatitis (ASH), hepatic fibrosis and cirrhosis [3]. ASH is characterized by the presence of inflammatory foci within the liver and the occurrence of macrophage activation and chemoattraction [4]. These mechanisms are closely associated with the production of reactive oxygen species (ROS) induced by ethanol and its metabolites, as well as the activation of innate immunity (e.g. the production of pro-inflammatory cytokines and chemokines) [5]. Therefore, administration of therapy directed against the initiation and progression of AFLD should at least target one or several key pathological events related to AFLD, such as steatosis, hepatic oxidative stress, and inflammation.

Zeaxanthin and luteins are the common carotenoid alcohols found in nature. As a potent free-radical scavenger as well as its hepatotropic and lipotropic distribution, zeaxanthin is considered as a promising hepato-protective agent against several liver diseases, including acute liver injury [6], liver cancer [7], and non-alcoholic fatty liver disease (NAFLD) [8], [9]. It is found that in the liver, supplementary intake of zeaxanthin could prevent hepatic oxidative stress and halt inflammation/fibrosis during the development of NAFLD, then reduces the chance of progressing to end-stage liver disease[8]. However, there is very limited information regarding the role and mechanism of zeaxanthin in AFLD. In addition, zeaxanthin is an important component of several natural antioxidant mixtures, such as in wolfberry, which is newly identified hepato-protective agent [10], [11]. Investigating the protective mechanism of ZD against AFLD may shed new direction on the pharmacological study of this compound. Considering the similarity of the pathological mechanisms between AFLD and NAFLD, the present study aimed to investigate the possible ameliorative and therapeutic effects of zeaxanthin (zeaxanthin dipalmitate) on ethanol induced fatty liver disease rat model.

## Materials and Methods

### Reagents and antibodies

**Figure 1:**
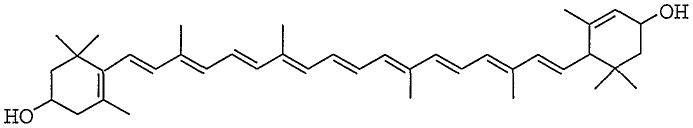
Structure of zeaxanthin.

Zeaxanthin dipalmitate (ZD) was purchased from Wako Pure Chemical Industries Ltd (Osaka, Japan). The structure of zeaxanthin is shown in Figure 1. Antibodies for cytochrome P450 2E1 (CYP2E1) and inhibitor of kappa B alpha (IκBα) were bought from Millipore (Billerica, MA) and Cell Signaling (Danvers, MA), respectively. Mitogen-activated protein kinase (MAPK) antibodies, including total p38 MAPK, phosphorylated p38 MAPK at Thr180/Tyr182, total c-Jun N-terminal kinase (JNK), phosphorylated JNK at Thr183/Tyr185, total extracellular signal-regulated kinase (ERK) and phosphorylated ERK at Tyr204 were purchased from Cell Signaling. Hoechset 33342, propidium iodide, SB203580 (p38 inhibitor), SP600125 (JNK inhibitor) and PD98059 (ERK inhibitor) were bought from Sigma-Aldrich (St. Louis, MO). RPMI-1640 cell culture medium, FBS and other cell culture consumables were obtained from Gibco (Life Technologies, Calsbad, CA).

### Animals and treatments

Twenty-four female Sprague-Dawley (SD) rats weighing from 180–200 g were obtained from the animal center of Zhejiang Experimental Animal Center. All experimental procedures were approved by the ethical committee of Zhejiang Ocean University. Before experiment, rats were adapted to the environment for one week with free access to regular chow and water. After that, rats were randomly and equally assigned to 4 groups (n = 6 per group) namely: (1) control group was fed with regular chow and water for 10 weeks. During this period, rats were orally fed with 5% Tween 80 in PBS which was the solvent used for ZD from the 5^th^ week to the 10^th^ week; (2) AFLD group was intragastrically fed with 4.0 g/kg ethanol diluted in distilled water in addition to oral regular chow and water for 10 weeks; (3) vehicle-ZD group was fed with regular chow and water for 10 weeks. During the period, rats were orally fed with 25 mg/kg body weight ZD (in 5% Tween 80-PBS) every day from the 5^th^ week to the 10^th^ week; and (4) AFLD+ZD group which received similar treatment as the AFLD group for the first five weeks and then orally fed with 25 mg/kg body weight ZD and intragastric administration of ethanol from the 5^th^ week to the 10^th^ week. Animal body weight was measured weekly to determine the effects of ethanol and ZD on body weight and to adjust the intragsatric ingestion of ethanol. Pilot study showed that oral feeding and intragastric administration of tween/PBS showed no obvious influence on the hepatic histology of rats (data not shown). Previous study showed that 25 mg/kg ZD could reduce hepatic fibrosis in rats at which we based the dose of ZD [12]. After 8 hrs of the last administration of ethanol and ZD, rats were euthanized with anoverdose of pentobarbitone. At week 10, blood samples were collected 1 hour after the ethanol administration in order to measure the blood alcohol level. Serum samples were obtained from whole blood by centrifugation at 3,000 rpm for 15 min. Liver samples were collected for histological examination, molecular and biochemical analyses.

### Liver tissue processing

Liver tissue samples were fixed in 10% phosphate-buffered formalin, and then embedded in paraffin blocks. Five-micrometer tissue sections were cut and stained with hematoxylin and eosin (H&E) for histological analysis under light microscope (Nikon, Japan). The mean area of necrosis was determined by dividing the sum areas of necrotic regions by the total number of fields per group [13].

### Serum biochemical assays

The serum alanine aminotransferase (ALT) and aspartate aminotransferase (AST) levels of rats were detected using the corresponding detection kits provided by Liaoning Taike Medical Science Company, Liaoning, China and according to the manufacture's protocols. Serum triglyceride (TG) and total cholesterol (TC) levels as well as liver tissue TG content were measured by commercial kits from Cayman Chemical (Cayman Chemical, Ann Arbor, MI). Serum ethanol level was measured with an alcohol dehydrogenase kit (Sigma), according to the protocol supplied by the manufacturer.

### Terminal deoxynucleotidyl transferase-mediated dUTP-nick end labeling (TUNEL) assay

To demonstrate the apoptosis in the liver, the TUNEL assay which detects 3′ hydroxyl ends in fragmented DNA as an early event in apoptotic cascade was used. After dewaxing and rehydration of the paraffin-embedded liver tissue sections, staining was performed using a TUNEL assay with the *in situ* cell death detection kit (Roche, Basel, Switzerland). The intensity of positive immunostaining of Fast Red of TUNEL was examined by light microscope (Olympus, Tokyo, Japan) and quantified by ImageJ software.

### Measurement of oxidative stress and lipid peroxidation in the liver

Liver tissue was homogenized in Tris-HCl buffer (20 mM, pH 7.4) and then centrifuged at 2500×g at 4°C for 10 min. Two hundred microliters of supernatant was analyzed for malondialdehyde (MDA) levels using a kit (Bioxytech MDA-586; Oxis Research, Foster City, CA) and the value was read at 586 nm. For the measurement of serum 8-isoprostane level, serum samples were quantified using the 8-isoprostane EIA kit (Cayman Chemical) according to manufacturer's instructions.

### Hepatic alcohol dehydrogenase (ADH) activity

Since ADH is one of the key enzymes responsible for the metabolism of ethanol in the liver, we measured its activity in different groups by using commercial kit (JianCheng Bioengineering Institute, Nanjing, China) according to the manufacturer's instructions.

### Cell culture and treatments

The BRL-3A cell line was supplied by the Cell Bank of Type Culture Collection of Chinese Academy of Sciences (Shanghai, China). It was cultured in RPMI-1640 with 10% (v/v) FBS at 37°C with 5% CO_2_ using a cell incubator. When cells reached a confluence of 60–70%, they were divided into 9 groups (n = 5) including: (1) control group (treated with 5% Tween 80 in PBS which was the solvent for ZD); (2) ethanol group (treated with 200 mM ethanol); (3) ethanol + ZD group (treated with 200 mM ethanol and 1 µM ZD); (4–6) inhibitor only groups [treated with p38 MAPK inhibitor – SB203580 (15 µM), JNK inhibitor – SP600125 (15 µM), and ERK inhibitor – PD98059 (15 µM), respectively]; (7–9) ethanol, ZD and MAPK inhibitor co-treatment groups (treated with 200 mM ethanol, 1 µM ZD, and corresponding MAPK inhibitors). The treatment duration was 24 hrs. Inhibitors for MAPK were added 2 hrs before the ethanol treatment and the specificity of each inhibitor was confirmed in a pilot study (data not shown). ZD was added 1 hr before the ethanol treatment. The dose selection of ZD was based on a previous study on carbon tetrachloride-induced rat hepatocyte injury model [14].

### MTT assay

Cell viability was evaluated by the conversion of 3-(4,5-Dimethylthiazol-2-yl)-2,5-diphenyltetrazolium bromide (MTT, Sigma-Aldrich) to a purple color product by cellular mitochondria. After drug treatment, cells from each group were washed by sterile PBS three times and then incubated with 5 mg/ml MTT for 3 hrs, and subsequently dissolved in dimethyl sulfoxide (DMSO). The absorbance of MTT was measured at 570 nm.

### Activities of caspase-3/7

Activities of caspases-3 and -7 from both liver tissue lysates and cell lysates were measured using Caspase-Glo 3/7 Assay Systems (Promega, Madison, WI) according to the user manual. The luminescence was read in a Glomax luminometer (Promega) and expressed as fold change in caspase 3/7 activity from control.

### RNA extraction and quantitative real-time PCR

Total RNA of each rat was extracted from the liver sample using RNeasy Mini Kit (Qiagen, Valencia, CA) and then quantified with the spectrophotometer (Beckman Coulter, Fullerton, CA). The reverse-transcription of total RNA was performed by SuperScript™ First-Strand Synthesis System (Life Technologies). The mRNA expression levels of catalase (CAT), superoxide dismutase 1 (SOD1), CYP2E1, Bcl-2, and Bax1 were measured by Perfect Real-time system on Applied Biosystems 7300 Real-time PCR system in triplicates. Parallel amplification of β-actin was used as the internal control. The reaction condition of all amplifications was: 10 min at 95°C, and 40 cycles of 15 sec at 95°C, 30 sec at 60°C, and 30 sec at 72°C. Primer sequences of target and house-keeping genes were listed in Table 1. Fold change of each gene was calculated by using the comparative Ct method. All quantitative PCR procedures, including the design of primers, validation of PCR environment, and quantification methods were performed according the MIQE guideline [15].

**Table 1:** Primer sequences for Real-time PCR.

Target gene	Direction	Primer sequence (5′-3′)
CAT	Sense	GAGGCAGTGTACTGCAAGTTCC
	Anti-sense	GGGACAGTTCACAGGTATCTGC
SOD1	Sense	GCAGGGCGTCATTCACTT
	Anti-sense	AGACTCAGACCACATAGGGA
Bcl-2	Sense	TGGGATACTGGAGATGAAGACT
	Anti-sense	CGACGGTAGCGACGAGA
Bax1	Sense	GGTTGCCCTCTTCTACTTTG
	Anti-sense	CAGCCACCCTGGTCTTG
CYP2E1	Sense	CCTTTCCCTCTTCCCATCC
	Anti-sense	AACCTCCGCACATCCTTCC
β-actin	Sense	GAGACCTTCAACACCCCAGCC
	Anti-sense	TCGGGGCATCGGAACCGCTCA

CAT, catalase; CYP2E1; cytochrome P450 2E1; SOD1; superoxide dismutase 1.

### Protein extraction and Western blotting

Liver samples were homogenized in lysis buffer (150 mM NaCl, 10 mM HEPES, pH 7.9, 1 mM EDTA, 0.6% NP-40, 0.5 mM PMSF, 1 µg/ml leupeptin, 1 µg/ml aprotonin, and 10 µg/ml trypsin inhibitor). Samples were then sonicated and incubated on ice for 30 minutes. Debris was removed by centrifugation at 10,000 rpm. Cellular total protein was extracted using a M-PER Mammalian Protein Extraction Reagent (Pierce, Rockford, IL) Protein concentrations of each sample were determined in order to calculate the loading amount. Samples were separated in a denaturing 10% polyacrylimide gel and transferred to a 0.1 µm pore nitrocellulose membrane. Non-specific binding sites were blocked with Tris-buffered saline (TBS; 40 mM Tris, pH 7.6, 300 mM NaCl) containing 5% nonfat dry milk (Cell Signaling) for 1 hour at room temperature. Membranes were then incubated with appropriate primary antibodies in TBS with 0.1% Tween 20 (TBST). Membranes were washed and incubated with secondary antibodies conjugated to horseradish peroxidase to show the bands with ECL buffer (GE Healthcare, UK). Parallel blotting of β-actin (Cell Signaling) was used as internal control.

### Enzyme-linked immunosorbent assay (ELISA) of cytokines and chemokines

ELISA measurements of TNF-α, IL-1β, IL-6, and MCP-1 protein expression levels were performed using kits from PeproTech (PeproTech Inc., Rocky Hill, NJ). Detailed procedures were performed according to the protocols provided by the manufacturer.

### NF-κB activity

The activation of transcriptional factor NF-κB was measured by using a NF-κB p65 transcription factor assay kit from Pierce, according to manufacturer's instructions. Nuclear protein from the liver tissue was extracted using a commercial kit from Pierce.

### Statistical analysis

All values were expressed as means and standard deviations. Statistical comparisons between groups were done using a variance (ANOVA) and followed by post hoc LSD tests. A p<0.05 was considered to be statistically significant (SPSS version 17.0., IBM Corporation, Armonk, New York).

## Results

### ZD treatment did not change blood alcohol level but increased rat body weight which was influenced by ethanol consumption

At week 10, blood alcohol levels 1 hour after ethanol administration were similar in the ZD-treated group (266.3±62.2 mg/100 ml) and the control group (294.4±69.1 mg/100 ml), indicating that supplementation with ZD did not alter the ethanol metabolism and any observed effects from ZD were not likely due to its effects on ethanol absorption. Before the start and 4 weeks after the experiment, there was no significant difference in rat body weights between each group. After 10-week 4.0 g/kg ethanol administration, rats from the AFLD group showed significantly decreased in body weight when compared with the control group (p<0.05). However, when ZD was co-treated from the 5^th^ week to the 10^th^ week, rats from the ALD+ZD group increased in body weight when compared with the AFLD group (p<0.05). Notably, vehicle-ZD treatment did not significantly alter the body weight of healthy rats (Table 2).

**Table 2:** The effect of ethanol consumption and ZD treatment on rat body weights.

Group	Body weights (g)		
	Week 1	Week 4	Week 10
Control	189.2±5.6^a^	223.9±10.7^a^	301.4±19.3^a^
AFLD	191.6±7.1^a^	217.6±11.8^a^	259.5±15.1^b^
Vehicle-ZD	190.5±6.0^a^	220.3±11.0^a^	298.6±21.2^a^
AFLD+ZD	186.9±5.7^a^	226.9±9.9^a^	277.5±14.2^c^

Values are means ± S.D.; n = 6.

Different letters (e.g. a and b) indicate significant change (at least p<0.05).

AFLD, alcoholic fatty liver disease; ZD, zeaxanthin dipalmitate.

### ZD treatment improved liver histology

**Figure 2:**
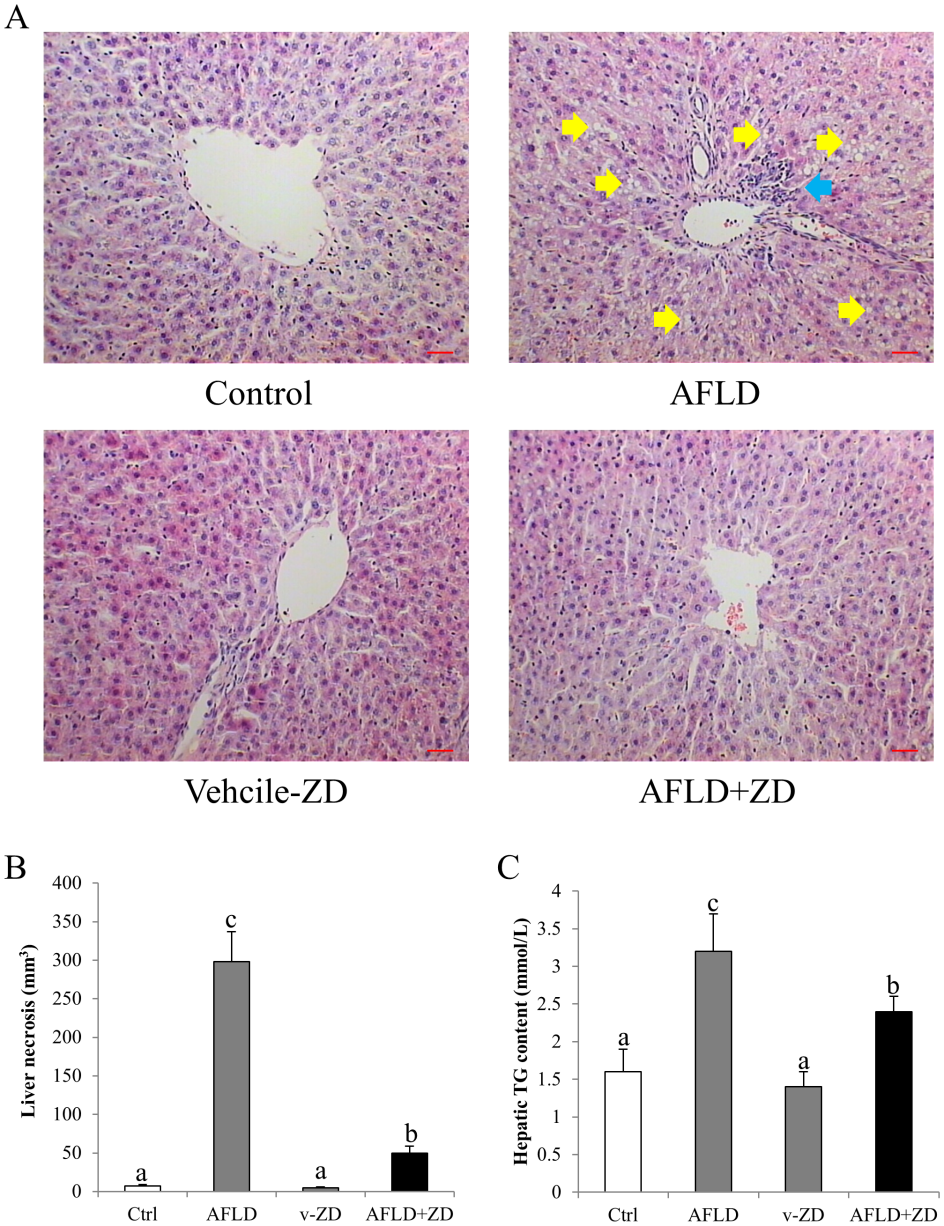
Ameliorative effects of zeaxanthin dipalmitate (ZD) on ethanol-induced hepatic steatosis and inflammation. (A) Representative histological changes of control group; alcoholic fatty liver disease (AFLD) group; vehicle-ZD group; and AFLD+ZD group after 10-week 4 g/kg ethanol administration with or without daily 25 mg/kg ZD administration from the 5^th^ week to the 10^th^ week. Tissue sections were stained with H&E (200*x* magnification). Yellow arrows indicate fat accumulation in the liver while blue arrow indicates typical inflammatory infiltration (Bar: 20 microns) (B) Liver necrotic area from each group. (C) Hepatic triglyceride (TG) content from each group. Different letters (e.g. a and b) indicate significant differences between groups (at least p<0.05). Data presented are expressed as Mean ± S.D. (n = 6).

When compared with the control group, the liver of ethanol-administered rats exhibited evidence of AFLD phenotypes, including fat droplet deposition and accumulation of inflammatory cells around the centrolobular vein (Figure 2A). Co-treatment with ZD significantly improved the hepatic pathology comparable to the control phenotype, with reduced number of lipid droplet and inflammatory foci (Figure 2A). The mean liver necrosis and hepatic TG content results further validated these findings (Figures 2B and 2C). Vehicle-ZD administration did not influence the liver morphology of the healthy rats. In line with the histolopathological results, ethanol treatment significantly increased the serum ALT level. ZD administration reduced it to the control level. AST level was not influenced by both ethanol and ZD treatments (Table 3). In addition, ZD treatment significantly inhibited ethanol-induced increase in serum TG and TC levels, which was consistent with the result of fat accumulation in the liver (Table 3 and Figure 2A).

**Table 3:** The effects of ethanol consumption and ZD treatment on rat serum markers.

Group	ALT (IU/L)	AST (IU/L)	TG (mmol)	TC (mmol)
Control	43.7±5.0^a^	133.2±13.5^a^	0.4±0.1^a^	1.1±0.2^a^
AFLD	65.2±6.6^b^	141.7±15.5^a^	0.7±0.1^b^	1.7±0.3^b^
Vehicle-ZD	44.5±5.2^a^	140.1±16.9^a^	0.4±0.2^a^	1.0±0.2^a^
AFLD+ZD	41.8±4.9^a^	138.6±17.2^a^	0.5±0.1^a^	1.2±0.2^a^

Values are means ± S.D.; n = 6.

Different letters (e.g. a and b) indicate significant change (at least p<0.05).

AFLD, alcoholic fatty liver disease; ZD, zeaxanthin dipalmitate; ALT, alanine aminotransferase; AST, aspartate aminotransferase; TG, triglyceride; TC, total cholesterol.

### ZD treatment attenuated hepatic oxidative stress

**Figure 3:**
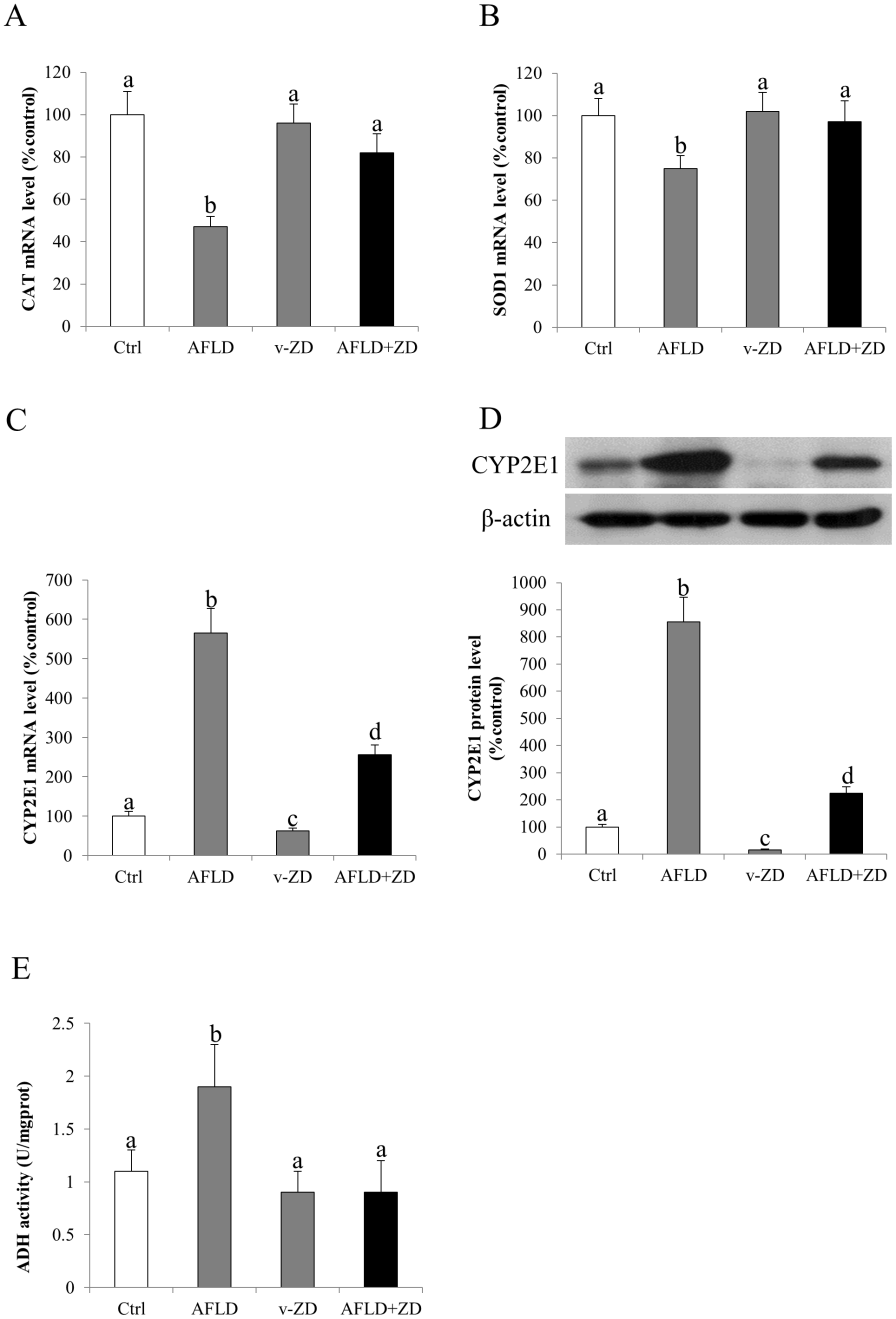
Zeaxanthin dipalmitate (ZD) attenuated hepatic oxidative stress and alcohol metabolizing enzyme induced by ethanol. Hepatic expression of (A) catalase (CAT) mRNA, (B) superoxide dismutase 1 (SOD-1) mRNA, (C) cytochrome P450 2E1 (CYP2E1) mRNA, (D) CYP2E1 protein, and (E) activity of alcohol dehydrogenase (ADH) after 10-week 4g/kg ethanol administration with or without daily 25 mg/kg ZD administration from the 5^th^ week to the 10^th^ week. Different letters (e.g. a and b) indicate significant differences between groups (at least p<0.05). Data presented are expressed as Mean ± S.D. (n = 6).

Oxidative stress is one of the most prominent pathological events during the progression of AFLD since it links lipid metabolism dysfunction and the occurrence of downstream inflammation and apoptosis [16]. To study the possible anti-oxidative stress effects of ZD, the mRNA expression of hepatic endogenous CAT and SOD1 was firstly evaluated. Chronic consumption of ethanol significantly reduced the mRNA expression level of both CAT and SOD1, indicating an impaired antioxidant status of the liver cells (Figures 3A and 3B). Administration of ZD recovered their expression levels comparable to the controls and without affecting the healthy rats (Figures 3A and 3B). CYP2E1 is one of the major mediators of oxidative stress during ethanol metabolism [17]. We also found that in our AFLD model, the mRNA and protein expression of CYP2E1 was markedly up-regulated by the ethanol administration, but it was significantly reduced by the after-treatment of ZD. Very interestingly, vehicle-ZD treatment down-regulated the basal mRNA and protein level of CYP2E1 (Figure 3C and 3D). In addition, in terms of end-products of oxidative stress, we measured the level of serum MDA and plasma 8-isoprostane in each group. Induction of AFLD significantly increased the formation level of MDA and 8-isoprostane, which was consistent with the results of CAT/SOD1 and CYP2E1. Intake of ZD significantly reduced these formations of the oxidative stress products (Table 4). In line with the oxidative stress markers, the activity of hepatic ADH was also induced by ethanol but it was suppressed by the after-treatment with ZD, which was also comparable to the control level (Figure 3E).

**Table 4:** **The** effects of ethanol consumption and ZD treatment on rat serum MDA and plasma 8-isoprostane levels.

Group	MDA (nmol/L)	8-isoprostane (pg/ml)
Control	7.5±1.2^a^	103.8±12.2^a^
AFLD	17.9±2.5^b^	255.6±29.4^b^
Vehicle-ZD	7.2±1.1^a^	97.4±11.0^a^
AFLD+ZD	11.3±2.1^c^	139.6±18.3^c^

Values are means ± S.D.; n = 6.

Different letters (e.g. a and b) indicate significant change (at least p<0.05).

AFLD, alcoholic fatty liver disease; ZD, zeaxanthin dipalmitate; MDA, malondialdehyde.

### Administration of ZD alleviated hepatic inflammation and apoptosis through MAPK pathway

**Figure 4:**
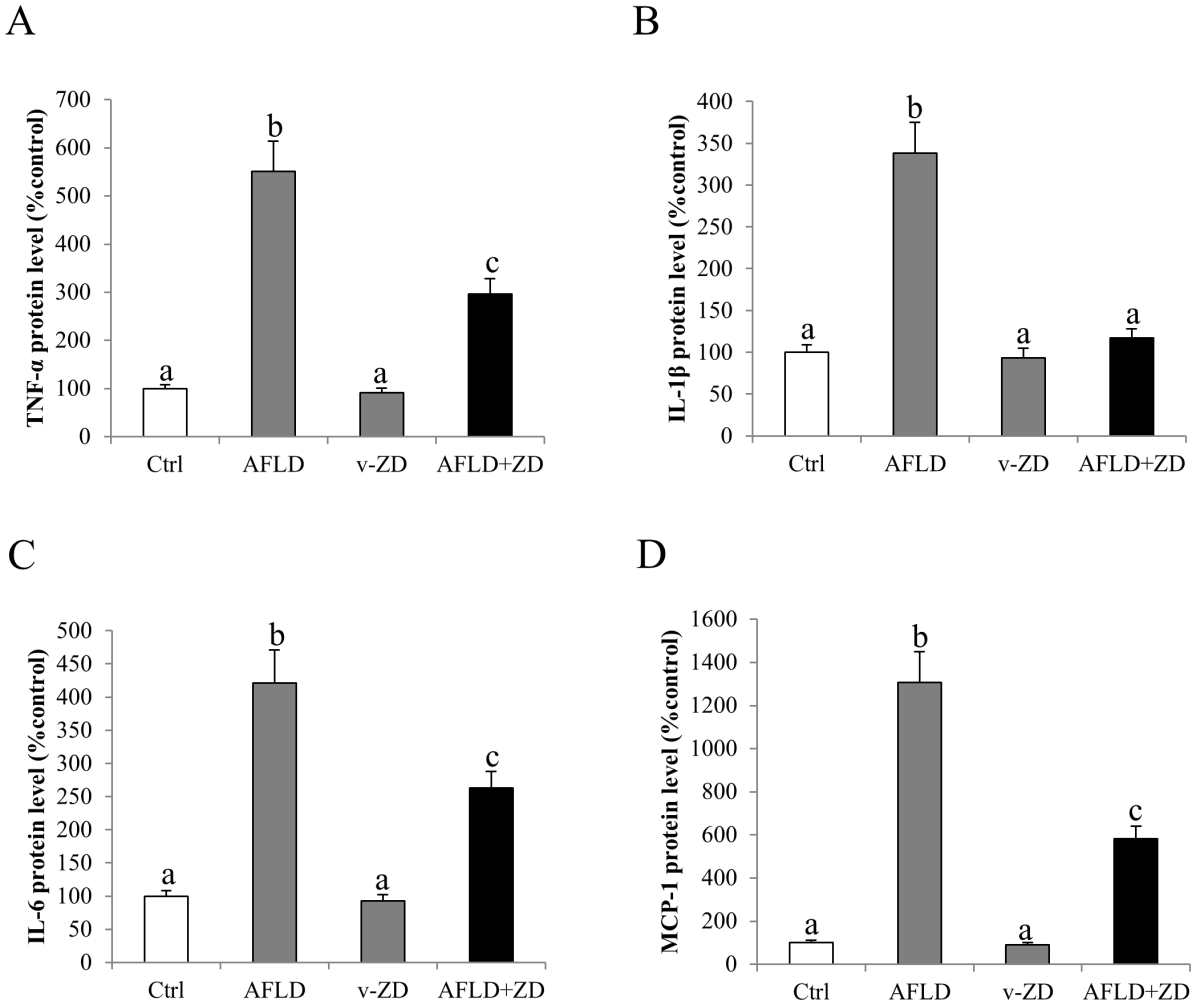
Zeaxanthin dipalmitate (ZD) attenuated hepatic inflammation induced by ethanol. Hepatic protein expression of (A) tumor necrosis factor alpha (TNF-α), (B) interleukin (IL)-1β, (C) IL-6, and (D) monocyte chemotactic protein (MCP)-1 after 10-week 4 g/kg ethanol administration with or without daily 25 mg/kg ZD administration from the 5^th^ week to the 10^th^ week. Different letters (e.g. a and b) indicate significant differences between groups (at least p<0.05). Data presented are expressed as Mean ± S.D. (n = 6).

To further investigate the beneficial effects of the therapeutic treatment ZD on AFLD, we then measured the change of protein expression of key markers of inflammation (TNF-α, IL-1β and IL-6) and chemoattraction (MCP-1) in each experimental group. Ten-week consumption of ethanol significantly up-regulated the expression levels of these proteins in the AFLD rat liver. Therapeutic treatment with ZD reduced or even abolished the influence of ethanol on these pro-inflammatory mediators (Figures 4A–4D).

**Figure 5:**
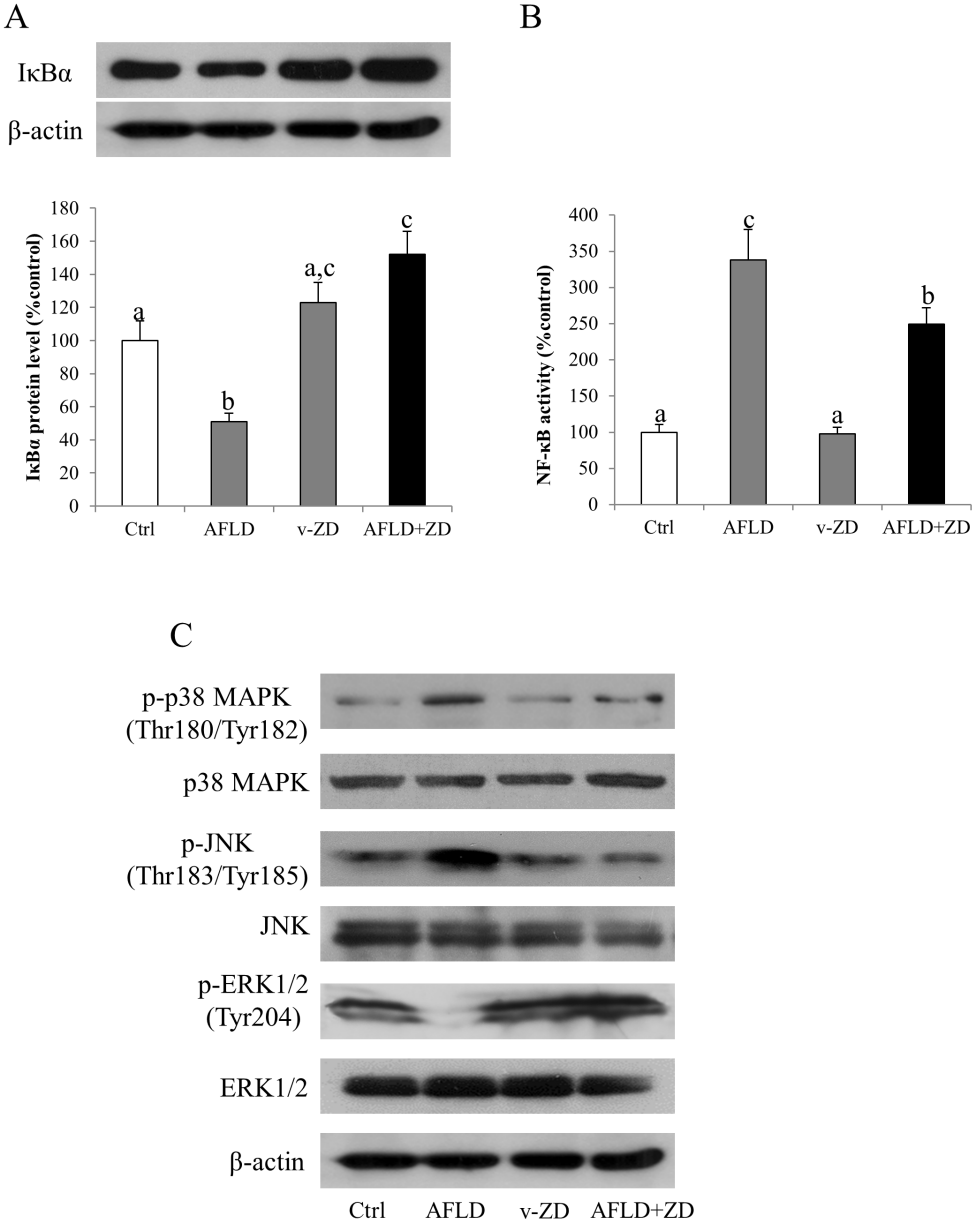
Zeaxanthin dipalmitate (ZD) played hepato-protective effects through modulating transcription factor and MAPK pathways. Hepatic expression of (A) cytosolic inhibitor of kappa B alpha (IκBα) protein, (B) nuclear factor kappa B (NF-κB) activity and (C) MAPK pathway members after 10-week 4 g/kg ethanol administration with or without daily 25 mg/kg ZD administration from the 5^th^ week to the 10^th^ week. Different letters (e.g. a and b) indicate significant differences between groups (at least p<0.05). Data presented are expressed as Mean ± S.D. (n = 6).

To investigate the underlying mechanisms of ZD-mediated hepatic improvements, we measured the expressional changes of key MAPK members in rats since they were heavily involved in the fatty liver diseases [10], [18]. Administration of ethanol significantly increased the phosphorylation of both p38 MAPK and JNK, but decreased the phosphorylation of ERK (Figure 5C). After-treatment with ZD abolished the influence of ethanol on phosphorylated p38 MAPK and JNK, but not the phosphorylation form of ERK. Both treatments of ethanol and ZD did not demonstrate any effects on the total form expression of p38 MAPK, JNK and ERK (Figure 5C).

**Figure 6:**
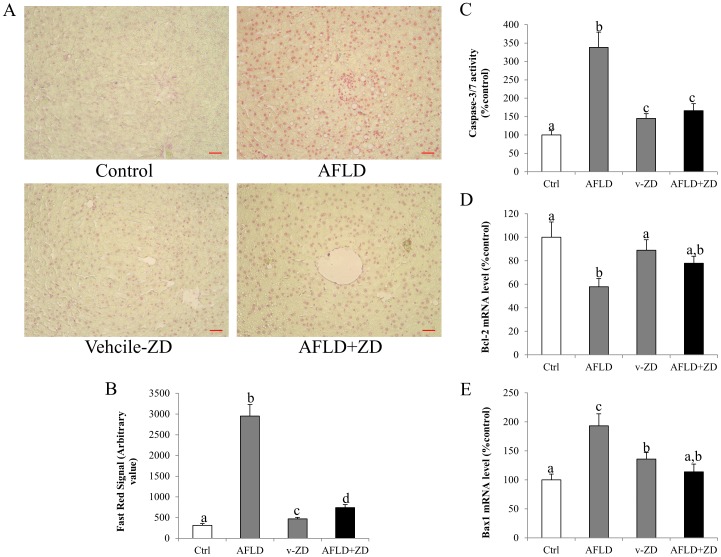
Zeaxanthin dipalmitate (ZD) attenuated hepatic apoptosis induced by ethanol administration. (A) Representative hepatic apoptotic changes of control group; alcoholic fatty liver disease (AFLD) group; vehicle-ZD group; and AFLD+ZD group after 10-week 4 g/kg ethanol administration with or without daily 25 mg/kg ZD administration from the 5^th^ week to the 10^th^ week. Tissue sections were stained with TUNEL assay kit (200*x* magnification; Bar: 20 microns). The results are further confirmed by the quantification of the Fast Red staining (B) and expression of caspase-3/7 activity (C) Bcl-2 mRNA (D) and Bax1 mRNA (E). Different letters (e.g. a and b) indicate significant differences between groups (at least p<0.05). Data presented are expressed as Mean ± S.D. (n = 6).

The intensity of Fast Red staining was pronounced in the AFLD rats but it was significantly reduced after the ZD treatment (Figures 6A and 6B). This observation was validated by the down-regulation of Bax1 mRNA level and the up-regulation of Bcl-2 mRNA level after the ZD treatment. The ALFD treated rats showed increased in Bax1 and decreased in Bcl-2 mRNA levels (Figures 6D and 6E). The activity of caspase 3/7 also exhibited similar trend to Bax1 (Figure 6C)

### In vitro investigation of the involvement of MAPK pathways

To further confirm whether the MAPK signaling pathways mediated the hepato-protective functions of ZD, rat hepatocyte cell line BRL-3A was pre-incubated with SB203580 (inhibitor of p38, 15 µmol/L), SP600125 (inhibitor of JNK, 15 µmol/L) or PD98059 (inhibitor of ERK, 15 µmol/L) before ethanol treatment and ZD intervention. Inhibitor single treatment did not affect the cell viability but PD98059 slightly induced expression of TNF-α, cellular apoptotic ratio and the activity of caspase-3/7 (Table 5) [19]. When MAPK inhibitors were pre-treated prior to the 24-h ethanol exposure, SB203580 and PD98059 did not significantly influence the effects of ethanol on cell viability, inflammatory and apoptotic responses. However, inhibition of JNK by SP600125 attenuated cellular apoptosis induced by ethanol (Table 5). Co-treatment with ZD partially reversed the effects of ethanol on cell viability, TNF-α expression, apoptotic ratio and caspase-3/7 activity. Such effects were abolished by the addition of inhibitors of p38 MAPK and ERK, but not JNK inhibitor (Table 5).

**Table 5:** **The** effects of ethanol, ZD, and MAPK inhibitors on rat hepatocyte BRL-3A viability, inflammatory and apoptotic marker levels.

Group	Viability (%control)	TNF-α (%control)	Percentage of apoptosis (%)	Caspase-3/7 (%control)
Control	100.0±9.3^a^	100.0±12.2^a^	3.4±0.5^a^	100.0±10.8^a^
Ethanol	62.5±7.1^b^	309.5±36.8^b^	37.4±5.2^b^	307.7±37.6^b^
Ethanol+ZD	77.8±8.0^c^	221.8±23.2^c^	19.9±2.4^c^	236.2±22.1^c^
ip38	89.7±8.9^a^	122.3±15.4^a^	5.1±1.1^a^	109.3±11.5^a^
iJNK	92.7±9.6^a^	112.0±10.7^a^	5.5±0.6^a^	98.9±8.8^a^
iERK	98.4±8.9^a^	140.5±17.0^d^	10.8±1.5^d^	144.3±15.0^d^
Ethanol+ip38	63.3±6.2^b^	283.6±30.4^b^	33.6±2.9^b^	268.0±33.9^b^
Ethanol+iJNK	70.7±6.0^b,c^	272.2±28.8^b^	30.1±3.4^f^	229.7±24.4^c^
Ethanol+iERK	61.1±4.5^b^	319.8±34.7^b^	35.9±5.0^b^	315.5±35.0^b^
Ethanol+ZD+ip38	60.1±6.9^b^	288.4±31.1^b^	35.7±6.6^b^	313.0±34.7^b^
Ethanol+ZD+iJNK	79.3±6.6^c^	196.9±22.2^c^	15.3±3.0^e^	208.3±21.9^c^
Ethanol+ZD+iERK	58.3±7.3^b^	275.8±35.0^b^	30.9±3.0^b^	288.4±36.6^b^

Values are means ± S.D., n = 5; Results of TNF-α, percentage of apoptosis, and caspase-3/7 are against corresponding cell viability.

Different letters (e.g. a and b) indicate significant change (at least p<0.05).

ZD, zeaxanthin dipalmitate; TNF-α, tumor necrosis factor alpha; ip38, inhibitor of p38 MAPK (SB203580); iJNK, inhibitor of JNK (SP600125); iERK, inhibitor of ERK (PD98059).

**Figure 7:**
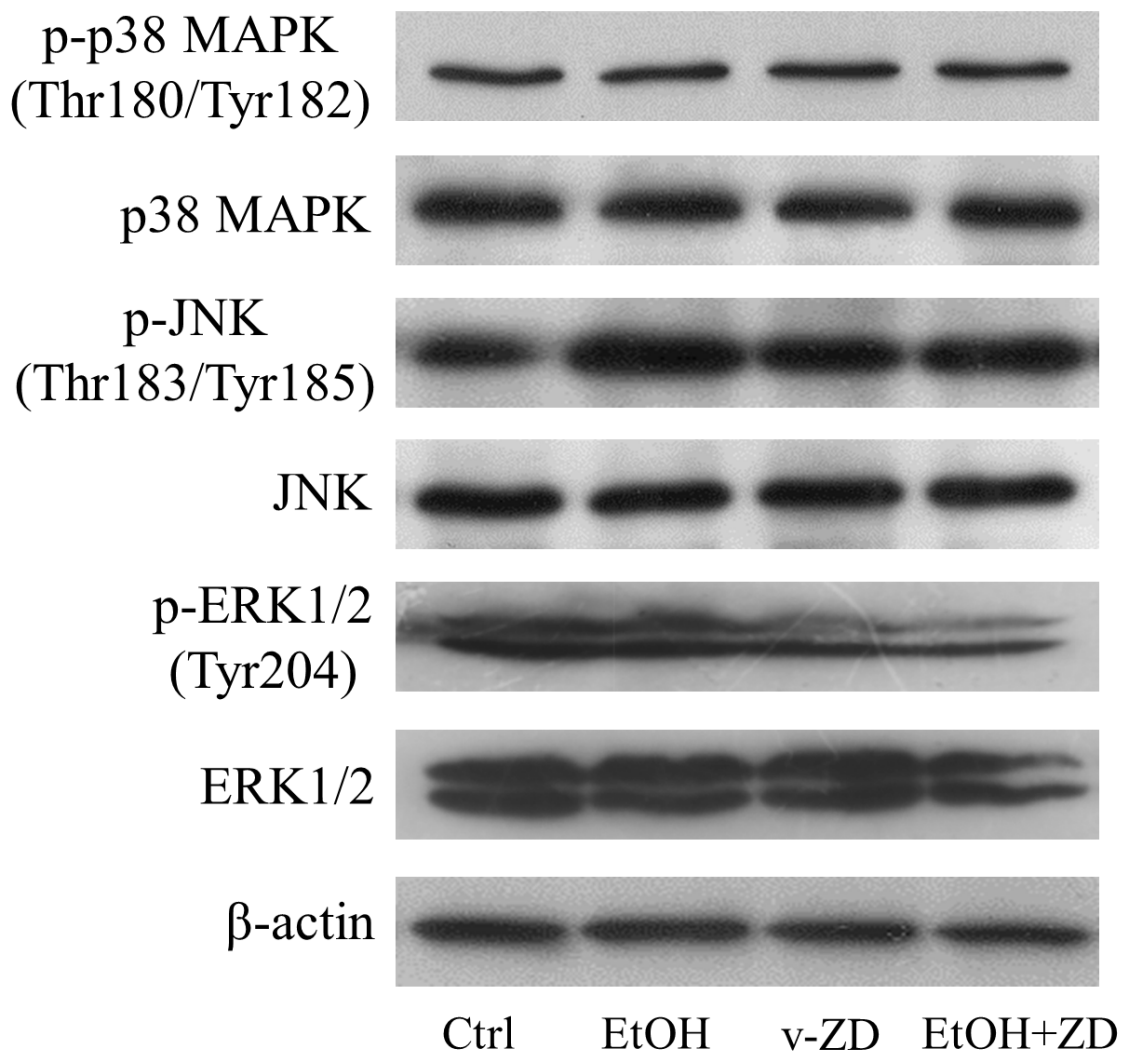
Effects of ethanol and zeaxanthin dipalmitate (ZD) on MAPK pathways in BRL-3A cells. Cellular changes of MAPK pathway members after 24-h 200 mM ethanol exposure with or without 1 µM ZD pre-treatment.

Next, we examined the effects of ethanol exposure and ZD co-treatment on the expression of phosphorylated and total forms of MAPK family members. It was shown that 24-h ethanol exposure did not change both the phosphorylated and the total forms of p38 MAPK and ERK, but it significantly promoted the phosphorylation of JNK. Such elevation was abolished by the addition of ZD (Figure 7). Co-treatment with ZD did not affect the phosphorylated and total forms of p38 MAPK but it slightly decreased the level of ERK (phosphorylated and total forms) (Figure 7).

## Discussion

AFLD is one of the biggest burdens to society, particularly in affluent regions. So the development of clinical therapy of AFLD has received much attention since abstinence is currently the only effective means to control the AFLD progression [20]. In recent years, emerging evidence suggests that several kinds of herbal derivatives may play protective and therapeutic roles against AFLD, such as wolfberry [21], resveratrol [22], green tea [23], and silibinin [24]. Due to their wide availability and low side-effect when administered within a reasonable dose range, the application of alternative herbal medicine is quite promising. However, for some herbal compounds like wolfberry, which consist of several monomers, although they possess potent hepato-protective properties, the identification of their active monomer(s) is still on the way [10]. In the current study, we demonstrated that 10-week administration of ethanol in rat caused typical AFLD features, including reduction of body weight, steatosis, hepatic oxidative stress, inflammation, chemoattraction and apoptosis. After treatment with zeaxanthine (ZD), from the 5^th^ week to the 10^th^ week of the experiment, it exhibited potent hepatoprotective, anti-oxidative stress, anti-inflammatory and anti-apoptotic properties. Such effects were achieved partially through the modulatory activity of transcription factor NF-κB and MAPK pathways including p38 MAPK and ERK, but not JNK. These results also demonstrated that ZD may partially account for the hepato-protective role of wolfberry and other herbal mixtures.

Serum ALT and AST are clinical biochemical markers for general hepatic cellular injury. Under healthy condition, they are contained within the hepatocytes. When there is a hepatic damage, either endogenous or exogenous, these enzymes will be released from hepatocytes into the circulation [25]. In our AFLD model, consumption of ethanol significantly increased the serum level of ALT but not AST, indicating the occurrence of hepatic injury, which was consistent with the histological results.

Addition of ZD not only reduced ALT content to the control level, but also significantly ameliorated the histopathological changes (e.g. number of fat deposition and inflammatory foci) (Figure 1 and Table 3). Also, the attenuation of steatosis in the liver by ZD was consistent with the reduction of the serum and hepatic TG and TC content. In addition, the reduction of inflammatory foci in the ZD-AFLD rat liver section was consistent with the down-regulation of expression level of hepatic pro-inflammatory markers and chemokines (Figure 3). It is now well recognized that inflammation is the key event which drives the transition from simple steatosis to hepatitis, both in AFLD and NAFLD [26]. The release of pro-inflammatory cytokines and chemokines are primarily from the hepatic resident macrophage–Kupffer cells [27]. Effective control of hepatic inflammation, either directly by inhibiting the activation of Kupffer cells or indirectly by suppressing transcription factor (e.g. NF-κB) may provide important beneficial effect on the progression and severity of AFLD. We also found that the activation of NF-κB by ethanol administration was significantly counteracted by ZD treatment, through the recovery of its cytosolic inhibitor IκBα, further ameliorating of the severity of ethanol-induced liver injury (Figures 4A and 4B).

The antioxidant properties of ZD had been shown in this study. Generation of hepatic oxidative stress from ROS and RNS is the direct consequence of ethanol metabolism. Since (1) CYP2E1 can produce ROS during its catalytic circle and (2) the activity of CYP2E1 can be elevated by chronic treatment with ethanol, CYP2E1 is considered as a major contributor to ethanol-induced hepatic oxidative stress [16], [17]. In this study, both mRNA and protein expression of CYP2E1 was induced by a 10-week administration of ethanol, leading to the increase of oxidative products (MDA and 8-isoprostane) and the decrease of endogenous antioxidant enzyme levels (CAT and SOD1). This result was in parallel with a previous study showing that CYP2E1 inhibitor chlormethiazole could partially protect the rat liver from ethanol-induced injury by inhibiting CYP2E1 activity *in vivo* [28]. Co-treatment with ZD effectively counteracted the induction of oxidative stress and restored the antioxidant defense of the cell, indicating its potent hepatic antioxidant property during AFLD development (Figure 2 and Table 4). The occurrence of oxidative stress and inflammation forms a positive pathogenic feedback loop. Therefore, the beneficial effects of ZD on both oxidative stress and inflammation strongly suggested its potential usage in therapy and prevention of hepatic injury.

Apoptosis is one of the most recognized features of AFLD [29]. In the current study, we confirmed that the number of apoptotic cells after ethanol treatment was significantly increased, both *in vivo* and *in vitro*. The induction of apoptosis was associated with the increase of caspase activity and the decrease of anti-apoptotic molecule (e.g. Bcl-2) (Figure 5). ZD treatment evidently reduced the apoptotic ratio through restoring Bcl-2 expression and reducing caspase activity. These data supported the beneficial effects of ZD on the liver by improving the anti-apoptotic response, which was consistent with a recent study showing the anti-apoptotic function of zeaxanthin through Bcl-2 and MAPK in human dermal papilla cells [30].

The core purpose of the current study was to investigate the potential molecular signaling pathways that were involved in the hepato-protective functions of ZD. We speculated the role of MAPK pathways in ZD hepato-protective effects since the polysaccharides portion of wolfberry potently alleviated NAFLD by modulating the MAPK pathways [10] and ZD was also one of the components of wolfberry [31]., We found that chronic ethanol treatment induced the activity (phosphorylated form) of p38 MAPK and JNK but decreased ERK. These results were consistent with previous findings from our wolfberry study [10]. Addition of ZD abolished the induction of p38 MAPK and JNK but not ERK. Notably, in the cell model, since (1) inhibitor of p38 MAPK or ERK alone did not ameliorate ethanol-induced cellular injury but effectively abolished the beneficial influence of ZD on the hepatic functions, and (2) ethanol or ZD incubation did not significantly regulate the phosphorylation and basal protein expression of p38 MAPK and ERK, it was reasonable to conclude that both p38 MAPK and ERK were closely associated with the ZD ameliorative functions against ethanol exposure-induced hepatocytes dysfunctions (Table 5 and Figure 7). Interestingly, JNK seemed to play a different role in ethanol-induced liver injury, because (1) ethanol exposure significantly elevated the phosphorylated level of JNK in BRL-3A cells and ZD co-treatment restored its basal level, (2) JNK inhibitor alone attenuated apoptosis, and (3) inhibition of JNK did not affect the alleviative effects of ZD. However, JNK was partially responsible for ethanol-induced cellular apoptosis but not involved in the protection from ZD co-treatment (Table 5 and Figure 7). This result is consistent with a previous report that p38 MAPK and ERK, but not JNK, are involved in the amelioration of carbon monoxide against ethanol-induced oxidative damage and inflammatory stress [32]. There is limited information about the signaling pathways regulated by zeaxanthin. Our study may provide impetus for further exploration of beneficial effects of zeaxanthin in the humans, not only confined in the liver.

In summary, we conclude that ZD therapeutically reduced ethanol-induced hepatotoxicity by (1) improving hepatic histology, (2) ameliorating steatosis, (3) increasing endogenous antioxidant enzyme level through CYP2E1-dependent pathway, (4) demising pro-inflammatory response and chemoattraction, and (5) attenuating hepatic apoptosis. These beneficial effects were partially regulated via the reduction of NF-κB activity and the modulation of p38 MAPK and ERK pathways. We suggest that ZD could be a good alternative herbal medicine that has great therapeutic and hepatoprotective properties for the treatment of liver diseases.
